# Synovitis detection in rheumatology: a systematic review and meta-analysis of contrast-enhanced ultrasound and magnetic resonance imaging

**DOI:** 10.1590/1516-3180.2025.3088.11112025

**Published:** 2026-04-10

**Authors:** Bruno Fernandes Barros Brehme de Abreu, Gabriella Simões Scarmagnan, Márcio Luís Duarte, Mayara Oliveira da Silva, Wagner Iared

**Affiliations:** I Radiologist, Santa Casa de Campo Grande, Campo Grande (MS), Brazil; Programa de Pós-Graduação em Saúde Baseada em Evidências, Escola Paulista de Medicina, Universidade Federal de São Paulo (PGSBE-EPM-Unifesp), São Paulo (SP), Brazil.; II Physiotherapist, Universidade Federal de Mato Grosso do Sul (UFMS), Campo Grande (MS), Brazil.; III Professor of Radiology, Universidade de Ribeirão Preto (UNAERP), Guarujá (SP), Brazil; Musculoskeletal Radiologist, Diagnósticos da América S.A, São Paulo (SP), Brazil.; IV Biomedicine, Programa de Pós-Graduação Interdisciplinar em Ciências da Saúde, Universidade Federal de São Paulo (PPGICSUnifesp), Santos (SP), Brazil; Clínica MegaImagem, Santos (SP), Brazil.; V Radiologist, Ultrasound Coordinator of Diagnósticos da América S.A., São Paulo (SP), Brazil.

**Keywords:** Ultrasonography, Magnetic resonance imaging, Synovitis, Contrast media, Microbubbles, Microbubble contrast, Rheumatoid arthritis, Arthritis

## Abstract

**BACKGROUND:**

Synovial tissue proliferation in the bare area of the joint is an early indicator of synovitis. Vascularization of the pannus helps differentiate between inactive and inflammatory processes, directly impacting therapeutic management. Synovitis can be diagnosed through clinical assessment, ultrasound, and magnetic resonance imaging (MRI); however, uncertainty remains regarding the optimal imaging modality.

**OBJECTIVE:**

This study aimed to determine the accuracy of ultrasonography with microbubble contrast and contrast-enhanced MRI in diagnosing synovitis, irrespective of its etiology. In addition, the study aimed to determine the joints that were most accurately assessed for synovitis using microbubble ultrasound.

**METHODS:**

Electronic searches were conducted in the Cochrane Library, MEDLINE, EMBASE, LILACS, SCOPUS, CINAHL, and Web of Science up to February 8, 2025, with additional screening of reference lists. Studies assessing diagnostic accuracy or detection rates of contrast-enhanced ultrasound (CEUS) and contrast-enhanced MRI for synovitis were included without restrictions on language or publication status. Two studies were selected after quality assessment using QUADAS-2, and eight studies were assessed using the RTI item bank methodology.

**RESULTS:**

Diagnostic accuracies of contrast-enhanced ultrasonography (87%) and contrast-enhanced MRI (87.7%) were comparable. For knee evaluation, CEUS showed a higher detection rate (93.8%) than MRI (82.9%). Across different joints and underlying diseases, the detection rates were 81.9% and 88.3% for contrast-enhanced MRI. In patients with rheumatoid arthritis, MRI demonstrated a higher detection rate (96.2%) compared with ultrasound (67.2%). These findings indicate a similar overall diagnostic performance, although the limited number of included studies restricted generalizability.

**CONCLUSION:**

CEUS demonstrated diagnostic accuracy comparable to contrast-enhanced MRI, except in patients with rheumatoid arthritis. Given its low cost, portability, and favorable safety profile, CEUS may serve as a useful screening or follow-up tool for synovitis, pending validation in larger multicenter studies.

## INTRODUCTION

 In the synovial joint, the surface of the articulating bones is covered by cartilage, except for a small region between the insertion of the fibrous capsule and the cartilage. In this area, known as the "bare area" of the joint, the bone is covered only by the synovium. The bone surface in this region, which is in direct contact with the synovial tissue without a protective cartilage layer, is highly susceptible to bone destruction induced by synovitis.^1,2^

 The proliferation of synovial tissue in this area is an early finding. Angiogenesis and hypervascularization, which result in pannus formation, are critical mechanisms that drive joint, cartilage, and bone destruction in the progression of rheumatoid arthritis. The presence of vascularization in the pannus can distinguish inactive from inflammatory processes and has significant implications for therapeutic management.^2^ Differentiating between inactive fibrotic synovial tissue and active pannus, as well as quantifying synovitis, is currently an important area of investigation.^3^

 Synovitis can be diagnosed through clinical evaluation, ultrasonography, and magnetic resonance imaging (MRI).^4^ Among these diagnostic tools, both ultrasound and MRI have advantages and disadvantages. However, no consensus is available on which imaging method offers superior accuracy in detecting and grading synovial inflammation across different joints and rheumatological diseases. This lack of comparative evidence warrants a systematic review of studies that directly assess both techniques. 

 The real-time capability of ultrasonography allows for the dynamic evaluation of joint movements, which can often help detect structural abnormalities. The advantages of ultrasound include its non-invasiveness, portability, cost-effectiveness, lack of ionizing radiation, and capability to be repeated as frequently as necessary, making it particularly useful for treatment monitoring. In contrast, ultrasound is operator-dependent, requiring highly experienced professionals with expertise in musculoskeletal anatomy and pathology as well as the ability to recognize artifacts that can often mimic lesions.^5^

 On a global scale, MRI evaluates all structures, including bones, and is more easily interpretable, does not utilize radiation, and is less operator-dependent. It is currently considered the gold standard for the diagnosis of synovitis.^6^ However, MRI is an expensive diagnostic technique not accessible to all patients, and has limitations in individuals with metallic implants, certain pacemakers, or claustrophobia.^7^

 Previous studies have compared these modalities in isolated contexts or in small series. However, a unified synthesis of available evidence comparing contrast-enhanced ultrasound (CEUS) and contrast-enhanced MRI across multiple joints remains limited. Thus, this systematic review and meta-analysis aimed to evaluate and compare the diagnostic accuracy of CEUS and MRI for synovitis, and to identify the optimal technique for specific joints and clinical scenarios. 

## METHODOLOGY

### Objectives

 This study aimed to determine the accuracy of CEUS using microbubbles to diagnose synovitis, regardless of etiology. In addition, the study compared the accuracy of CEUS with microbubbles and contrast-enhanced MRI and determined which method was superior for diagnosing synovitis. Finally, the study aimed to identify which joints showed better accuracy for the detection of synovitis than using CEUS. 

### Study design

 A systematic review of diagnostic accuracy studies was conducted using the *Cochrane Diagnostic Reviewer’s Handbook* version 5.1. 

### Inclusion criteria

 Studies evaluating the diagnostic accuracy and detection rates of CEUS and MRI for synovitis were included, and specifically all studies regardless of publication status; no language restrictions were included in the analysis. The review was registered in the OPENSCIENCE database with the registration number DOI: 10.17605/OSF.IO/96HDC. 

### Participants

 Patients of all ages and sexes with clinically confirmed synovitis, irrespective of disease severity or duration. 

### Tests evaluated

 CEUS assessing synovial enhancement with microbubble contrast agents and contrast-enhanced MRI evaluating synovial enhancement with gadolinium contrast agents were compared. 

### Reference standard

 The included studies described clinical and laboratory diagnoses of arthritis or osteoarthritis based on the established Rheumatology Society criteria. 

### Study selection and data extraction

 Eligible publications were selected based on relevant articles or abstracts from indexed peer-reviewed journals. Independent selection by two authors. In cases of disagreement, a third reviewer was consulted. Data were extracted using a standardized form that included methods, participant characteristics, outcomes, and results. 

### Methodological quality assessment

 Eligible studies with control groups were assessed using the QUADAS-2 (Quality Assessment of Diagnostic Accuracy Studies) tool.^8^ The tool comprises four domains: patient selection, index test, reference standard, flow, and timing. Each domain was evaluated as having a high, low, or unclear risk of bias. The applicability of the first three domains was evaluated using the following classifications: high, low, or unclear. The Signaling questions supported the domain evaluations. 

 Eligible studies were assessed using the RTI Item Bank, a tool focused on evaluating bias and precision.^9,10^ This tool comprises 29 multiple-choice questions covering 11 domains: sample definition and selection, interventions/exposure; outcomes; blinding; data robustness, follow-up; comparative analysis, interpretation; and reporting. 

 Responses included "Yes," "No," "Partially," "Cannot Determine," and "Not Applicable." 

### Search methods for study identification

 Electronic Searches were conducted in Cochrane Library, MEDLINE, EMBASE, LILACS, SCOPUS, CINAHL, and WEB OF SCIENCE up to February 8, 2025; Reference lists of the included studies and key reviews on the topic were also checked. Manual Searches were performed in reference lists of identified articles. 

 The search strategy included MeSH terms: "synovitis," "ultrasonography," "microbubbles," "contrast media," and "magnetic resonance imaging," as detailed in **Table 1**. 

**Table 1:** Search strategy

**Database**	**Search strategy**
Cochrane Library	#1: MeSH descriptor: [Synovitis] explode all trees
	#2: MeSH descriptor: [Ultrasonography] explode all trees
	#3: MeSH descriptor: [Microbubbles] explode all trees
	#4: MeSH descriptor: [Contrast media] explode all trees
	#5: MeSH descriptor: [Magnetic Resonance Imaging] explode all trees
	#6: #1 AND #2 AND #3 OR #4 AND #5
Medline	#1: "Synovitis"[MeSH] OR (Synovitides) OR (Synovial Plica Syndrome) OR (Plica Syndrome, Synovial) OR (Plica Syndrome) OR (Synovial Hypertrophy) OR (Hypertrophies, Synovial) OR (Hypertrophy, Synovial) OR (Synovial Hypertrophies) OR (Synovial Thickening) OR (Synovial Thickenings) OR (Thickening, Synovial) OR (Thickenings, Synovial)
	#2: "Ultrasonography"[MeSH] OR (Echotomography) OR (Diagnostic Ultrasound) OR (Diagnostic Ultrasounds) OR (Ultrasound, Diagnostic) OR (Ultrasounds, Diagnostic) OR (Sonography, Medical) OR (Medical Sonography) OR (Ultrasound Imaging) OR (Imaging, Ultrasound) OR (Imagings, Ultrasound) OR (Ultrasound Imagings) OR (Echography) OR (Ultrasonic Imaging) OR (Imaging, Ultrasonic) OR (Echotomography, Computer) OR (Computer Echotomography) OR (Tomography, Ultrasonic) OR (Ultrasonic Tomography) OR (Diagnosis, Ultrasonic) OR (Diagnoses, Ultrasonic) OR (Ultrasonic Diagnoses) OR (Ultrasonic Diagnosis)
	#3: "Microbubbles"[Mesh] OR (Microbubble) OR (Colloidal Gas Aphrons) OR (Aphron, Colloidal Gas) OR (Aphrons, Colloidal Gas) OR (Colloidal Gas Aphron) OR (Gas Aphron, Colloidal) OR (Gas Aphrons, Colloidal)
	#4: "Contrast media"[MeSH] OR (Media, Contrast) OR (Contrast Agent) OR (Agent, Contrast) OR (Contrast Materials) OR (Materials, Contrast) OR (Contrast Agents) OR (Agents, Contrast) OR (Contrast Material) OR (Material, Contrast) OR (Radiocontrast Media) OR (Media, Radiocontrast) OR (Radiocontrast Agent) OR (Agent, Radiocontrast) OR (Radiocontrast Agents) OR (Agents, Radiocontrast) OR (Radiopaque Media) OR (Media, Radiopaque)
	#5: "Magnetic Resonance Imaging"[MeSH] OR (Imaging, Magnetic Resonance) OR (NMR Imaging) OR (Imaging, NMR) OR (Zeugmatography) OR (Tomography, MR) OR (Tomography, NMR) OR (MR Tomography) OR (NMR Tomography) OR (Tomography, Proton Spin) OR (Proton Spin Tomography) OR (Magnetization Transfer Contrast Imaging) OR (MRI Scans) OR (MRI Scan) OR (Scan, MRI) OR (Scans, MRI) OR (fMRI) OR (MRI, Functional) OR (Functional MRI) OR (Functional MRIs) OR (MRIs, Functional) OR (Functional Magnetic Resonance Imaging) OR (Magnetic Resonance Imaging, Functional) OR (Imaging, Chemical Shift) OR (Chemical Shift Imagings) OR (Imagings, Chemical Shift) OR (Shift Imaging, Chemical) OR (Shift Imagings, Chemical) OR (Chemical Shift Imaging)
	#6: #1 AND #2 AND #3 OR #4 AND #5
EMBASE	#1: ‘synovitis’/exp OR ‘immune synovitis’ OR ‘inflammation, synovia’ OR ‘synovia inflammation’ OR ‘synovial disease’ OR ‘synovial inflammation’ OR ‘synovitis’ OR ‘synovium inflammation’ OR ‘toxic synovitis’ OR ‘villous synovitis’
	#2: ‘echography’/exp OR ‘diagnostic ultrasonic examination’ OR ‘diagnostic ultrasonic imaging’ OR ‘diagnostic ultrasonic method’ OR ‘diagnostic ultrasound’ OR ‘doptone’ OR ‘duplex echography’ OR ‘echogram’ OR ‘echographic evaluation’ OR ‘echography’ OR ‘echoscopy’ OR ‘echosound’ OR ‘high resolution echography’ OR ‘scanning, ultrasonic’ OR ‘sonogram’ OR ‘sonographic examination’ OR ‘sonographic screening’ OR ‘sonography’ OR ‘ultrasonic detection’ OR ‘ultrasonic diagnosis’ OR ‘ultrasonic echo’ OR ‘ultrasonic examination’ OR ‘ultrasonic scanning’ OR ‘ultrasonic scintillation’ OR ‘ultrasonogram’ OR ‘ultrasonographic examination’ OR ‘ultrasonographic screening’ OR ‘ultrasonography’ OR ‘ultrasound diagnosis’ OR ‘ultrasound scanning’
	#3: ‘microbubbles’
	#4: ‘contrast medium’/exp OR ‘contrast agent’ OR ‘contrast dye’ OR ‘contrast material’ OR ‘contrast media’ OR ‘contrast medium’ OR ‘radiocontrast medium’ OR ‘radiography contrast medium’ OR ‘roentgen contrast medium’
	#5: ‘nuclear magnetic resonance imaging’/exp OR ‘mri’ OR ‘nmr imaging’ OR ‘imaging, magnetization transfer’ OR ‘magnetic resonance imaging’ OR ‘magnetic resonance tomography’ OR ‘magnetization transfer imaging’ OR ‘mr imaging’ OR ‘nuclear magnetic resonance imaging’
	#6: #1 AND #2 AND #3 OR #4 AND #5
LILACS	#1: mh:” Sinovite” OR (Synovitis) OR (Sinovitis) OR (Synovite) OR (Espessamento Sinovial) OR (Hipertrofia Sinovial) OR (Síndrome da Prega) OR (Síndrome da Prega Sinovial) OR (mh: C05.550.870$)
	#2: mh: “Ultrassonografia” OR (Ultrasonografía) OR (Ultrasonography) OR (Ecografia) OR (Ecotomografia Computador) OR (Sonografia Médica) OR (Ecografia Médica) OR (Tomografia Ultrassônica) OR (Diagnóstico Ultrassom) OR (Imagem Ultrassônica) OR (Imagem Ultrassonográfica) OR (Imagem Ultrassom) OR (Imagem Ultrassom) OR (Ecotomografia) OR (mh:E01.370.350.850$)
	#3: mh:”Microbolhas” OR (Microbubbles) OR (Microburbujas) OR (Microbulles) OR (Microbolhas de Gás) OR (mh:E07.553$)
	#4: mh:”Meios de Contraste” OR (Contrast Media) OR (Medios de Contraste) OR (Produits de contraste) OR (Agente de Contraste) OR (Material de Contraste) OR (Meio Radiopaco) OR (Meio de Contraste) OR (Meios Radiopacos) OR (mh:D27.505.259.500$) OR (mh:D27.720.259$)
	#5: mh:”Imageamento por Ressonância Magnética” OR (Magnetic Resonance Imaging) OR (Imagen por Resonancia Magnética) OR (Imagerie par résonance magnétique) OR (IRM Funcional) OR (IRMf) OR (Imageamento Contrastado por Transferência de Magnetização) OR (Imageamento de Ressonância Magnética) OR (Imageamento de Spin-Eco) OR (Imageamento por Chemical Shift) OR (Imageamento por Ressonância Magnética Funcional) OR (Imagem Contrastada por Transferência de Magnetização) OR (Imagem de Ressonância Magnética) OR (Imagem de Spin-Eco) OR (Imagem por Chemical Shift) OR (Imagem por RMN) OR (Imagem por Ressonância Magnética) OR (Imagem por Ressonância Magnética Funcional) OR (Ressonância Magnética com Seqüências em Equilíbrio Estável) OR (Tomografia do Spin do Próton) OR (Tomografia por RM) OR (Tomografia por RMN) OR (Varreduras por IRM) OR (mh:E01.370.350.825.500$)
	#6: #1 AND #2 AND #3 OR #4 AND #5
SCOPUS	#1: Ultrasonography
	#2: Microbubble
	#3: Contrast media
	#4: Magnetic resonance imaging
	#5: Synovitis
	#6: #1 AND #2 OR #3 AND #4 AND #5
CINAHL	#1: Ultrasonography or ultrasound or sonography or echography
	#2: Microbubble
	#3: Contrast media or contrast medium or contrast agent
	#4: Magnetic resonance imaging or mri or mri scan
	#5: Synovitis
Web of Science	#1: Ultrasonography
	#2: Microbubble
	#3: Contrast media
	#4: Magnetic resonance imaging
	#5: Synovitis
	#6: #1 AND #2 OR #3 AND #4 AND #5

### Statistical analysis and data synthesis

 The study data were synthesized into 2 × 2 contingency tables, categorizing true positives, false positives, true negatives, and false negatives as absolute values. For the detection rate, the synovitis region was evaluated. All analyses were performed using RevMan 5.3. Diagnostic methods (CEUS and MRI) were compared based on the available patient data to minimize bias. No formal heterogeneity analysis (I² statistics) or publication bias tests (e.g., funnel plot) were conducted because of the small number of included studies. This limitation was considered when interpreting our findings. 

## RESULTS

**Figure 1:**
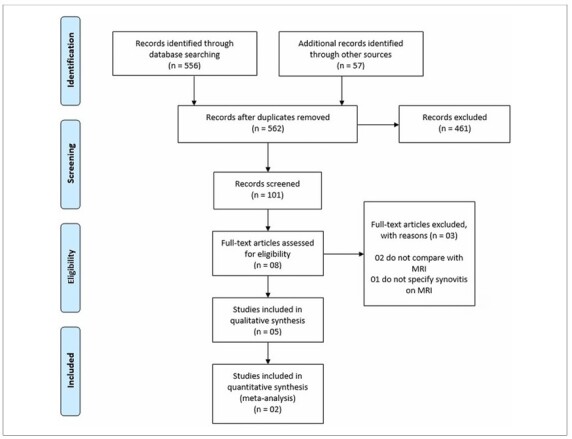
PRISMA diagram showing study selection.

 A total of 613 studies related to the topic were found in the literature searches, and eight studies that met the inclusion criteria were selected (**Figure 1**). One study^6^ did not specify whether synovitis was detected in the examinations, nor did it distinguish it from joint effusion and bone erosion. Two studies^4 ,11^ did not specify the exact number of patients who underwent examinations that showed synovitis on diagnostic tests. These studies were excluded from this systematic review. Additional data were requested by email; however, we did not receive any responses. In total, five studies were included in the final analysis (n = 235 patients), representing a small but methodologically consistent sample (**Table 2**).^12-16^

**Table 2:** Summary of the main characteristics of the included studies

**Study**	**Year**	**Sample (n patients)**	**Control group**	**Joints evaluated**	**Underlying diseases**	**Imaging methods compared**	**Main outcomes**
Magarelli et al.^14^	2001	40	No	Knee, wrist, ankle, elbow	RA, psoriatic arthritis, JRA, gout, Behçet’s	CEUS vs MRI	CEUS detected 93% of knees with synovitis
Szkudlarek et al.^13^	2003	42	Yes	Metacarpophalangeal	RA	CEUS vs MRI	MRI detected 96%, CEUS 67%
Wamser et al.^16^	2003	33	No	Shoulder	RA	CEUS vs MRI	Marked discrepancy in shoulder detection rates
Song et al.^12^	2008	41	Yes	Knee	Osteoarthritis	CEUS vs MRI	CEUS 93.8%, MRI 82.9%
Stramare et al.^15^	2013	79	No	Multiple joints	RA	CEUS vs MRI	Comparable accuracy

CEUS, contrast-enhanced ultrasound; RA, rheumatoid arthritis; JRA, juvenile rheumatoid arthritis; MRI, magnetic resonance imaging.

**Figure 2:**
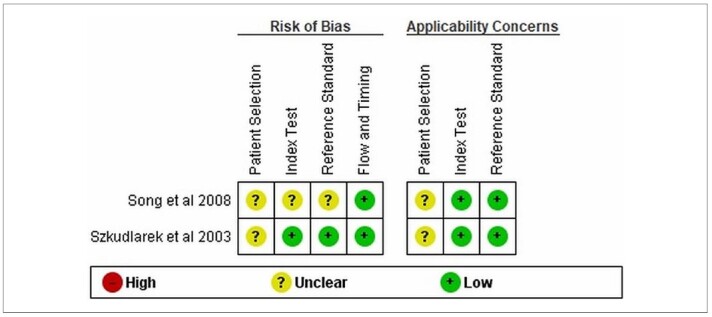
Tables assessing the risk of bias and the applicability of the quality of studies with a control group using the QUADAS-2 tool.

 Two studies were conducted with control groups, Song et al.^12^ and Szkudlarek et al.^13^ , thus allowing the evaluation of accuracy (**Figure 2**). Studies that did not have a control group (Magarelli et al.,^14^ Stramare et al.,^15^ and Wamser et al.^16^ ) were used to evaluate the detection rate of each method in conjunction with studies with a control group. This heterogeneity in the study design limited the statistical combination of the results but provided complementary evidence for comparative analysis. 

### Joints evaluated

 Three studies evaluated the knees,^12 ,14,15^ three evaluated the shoulders,^14-16^ two evaluated the elbows,^14,15^ two evaluated the wrists,^14,15^ two evaluated the metacarpophalangeal joints,^13,15^ one evaluated the carpal joints,^14^ one evaluated the interphalangeal joints,^15^ and one evaluated the ankles.^14^ However, the study by Stramare^15^ did not report the findings of synovitis in each joint, either by ultrasound or MRI, and the study by Magarelli^14^ did not report the synovitis findings in all joints separately by MRI, except for the knees, wrists, ankles, and elbows. 

 The knees had complete data from two studies, Magarelli et al.^14^ and Song et al.,^12^ totaling 106 knees evaluated. 

**Figure 3:**
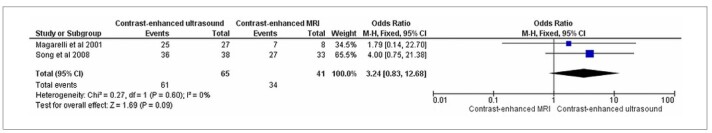
Comparison for knees: contrast-enhanced ultrasound versus contrast-enhanced MRI.

 Of the 65 knees assessed by CEUS, 61 showed synovitis, with a detection rate of 93.8%. Of the 41 knees evaluated using contrast-enhanced MRI, 34 showed synovitis, with a detection rate of 82.9% (**Figure 3**). 

### Underlying diseases evaluated

 Three studies evaluated synovitis in rheumatoid arthritis,^13,15 ,16^ and one in osteoarthritis.^12^ Magarelli et al.^14^ evaluated rheumatoid arthritis, psoriatic arthritis, chronic juvenile rheumatoid arthritis, gout, septic arthritis, and Behçet’s disease, reporting synovitis findings on CEUS, but not on MRI. 

**Figure 4:**
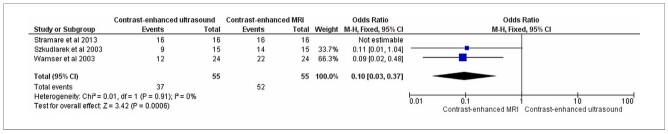
Comparison for rheumatoid arthritis: contrast-enhanced ultrasound versus contrast-enhanced MRI.

 Rheumatoid arthritis had complete data in three studies: Stramare et al.,^15^ Szkudlarek et al.,^13^ and Wamser et al.,^16^ with a total of 108 patients. Of the 55 patients evaluated by CEUS, 37 showed synovitis, with a detection rate of 67.2%. Of the 53 patients evaluated using contrast-enhanced MRI, 51 showed synovitis, with a detection rate of 96.2% (**Figure 4**). 

### Evaluation of synovitis independently of the joint and underlying disease

**Figure 5:**
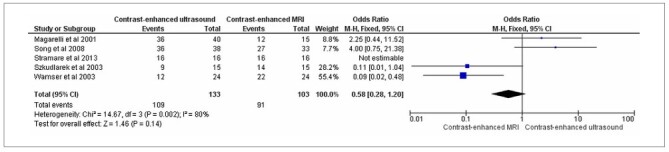
Comparison for synovitis regardless of the joint and underlying disease: contrast-enhanced ultrasound versus contrastenhanced MRI.

 The total number of patients evaluated was cited in five studies: Magarelli et al.,^14^ Song et al.,^12^ Stramare et al.,^15^ Szkudlarek et al.,^13^ and Wamser et al.,^16^ with 235 evaluated patients. Of the 133 patients evaluated by CEUS, 109 had synovitis (detection rate, 81.9%). Of the 103 patients evaluated using CE-MRI, 91 (88.3%) had synovitis (**Figure 5**). 

 Despite similar rates, the limited number of studies and heterogeneous populations precluded definitive statistical comparisons. 

### Accuracy evaluation

 Two studies were conducted with contrast enhancement, with a control group of patients affected by synovitis (Song et al.^12^ and Szkudlarek et al.^13^ ), thus allowing for the evaluation of accuracy. 

**Figure 6:**
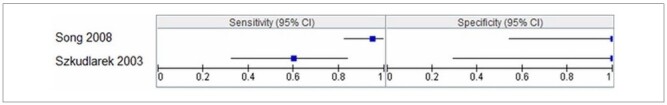
Accuracy Graph for synovitis: contrast-enhanced ultrasonography.

 The evaluation of CEUS in patients with synovitis showed 84.9% sensitivity and 100% specificity, with a 95% confidence interval (95% CI), p < 0.05, and 87% accuracy (**Figure 6**). 

 Two studies, Song et al.^12^ and Szkudlarek et al.,^13^ were conducted using contrast-enhanced MRI, with a control group of patients affected by synovitis, and thus, allowed for the evaluation of accuracy. 

**Figure 7:**
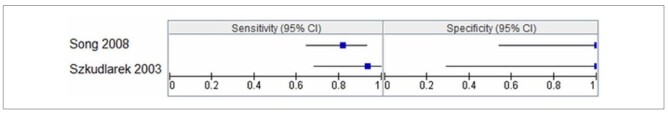
Accuracy Graph for synovitis: contrast-enhanced magnetic resonance imaging.

 The evaluation of contrast-enhanced MRI in patients with synovitis showed 85.4% sensitivity and 100% specificity, with a 95% CI and p < 0.05, and 87.7% accuracy (**Figure 7**). 

 These results indicate a comparable diagnostic performance between the two modalities. However, the small sample size and variations in joint types should be considered when interpreting these values. 

## DISCUSSION

 The diagnostic accuracy of the evaluated methods for synovitis showed no significant differences 87% for CEUS versus 87.7% for MRI. Regarding synovitis detection in patients with rheumatoid arthritis, contrast-enhanced MRI was superior (96.2%) to contrast-enhanced ultrasonography (67.2%). 

 Only one joint, the knee, presented data from different studies, which allowed for its evaluation. The synovitis detection rate in the knees was higher with CEUS (93.8%) than with CEMRI (82.9%). However, owing to the small number of joints evaluated, the forest plot did not show any differences between the diagnostic tests. 

 Evaluation of synovitis, independent of the joint and underlying disease, also showed a difference in detection rates between contrast-enhanced ultrasonography (81.9%) and contrast-enhanced MRI (88.3%). Both methods showed good sensitivity, with no significant difference between them (CEUS, 84.9%; contrast-enhanced MRI, 85.4%). Both methods showed 100% specificity. However, these findings should be interpreted with caution as they are based on a limited number of heterogeneous studies, including variations in disease type, joint distribution, and imaging protocols. 

 In terms of detecting inflammation, both ultrasound and MRI can detect more cases of synovitis than physical examination.^17^ The benefit of ultrasound as a complement to physical examination is influenced by the ability of subclinical synovitis to predict disease progression. Owing to their three-dimensional acquisition, both ultrasound and MRI are more sensitive than conventional radiography in detecting damage from erosion and early signs of erosion.^17^

 Osteoarthritis is a leading cause of labor disability worldwide, with synovitis being the earliest inflammatory sign.^18^ Several arthroscopy studies have also shown that synovitis is a common finding in osteoarthritis and is associated with disease progression.^19-21^ In 2007, rheumatoid arthritis was the fourth disease with the greatest budgetary impact on the Unified Health System in Brazil, consuming 10.4% of the resources.^22^ With these data, using CEUS instead of contrast-enhanced MRI for synovitis diagnosis could lead to significant savings, as ultrasound is less expensive than MRI (US$ 86.1, or R$ 438.3 for ultrasound; US$ 181.95, or R$ 926.3 for MRI), and microbubble contrast is also slightly less expensive than gadolinium (US$ 42.6, or R$ 216.7 for microbubble contrast; US$ 42.9, or R$ 218.5 for gadolinium).^23^ Although these values of rheumatoid arthritis reflect primarily Brazilian cost estimates, international comparisons show a similar cost gap favoring ultrasound. Thus, CEUS, being more accessible and performing faster, could reduce the waiting time for diagnosis and treatment, leading to lower hospitalization costs and reduced economic impact of patient disability. 

 After an intravenous bolus injection of microbubble contrast, the distribution can be monitored in real time, allowing for the delineation of structures and temporal evaluation. It is important to note that the adverse event rate is close to zero (1:10,000 compared with iodinated contrast agents, 1–12:100, and gadolinium contrast agents occurring in 0.04–0.3% of administrations, of which 0.4–9% are severe).^24-26^ These adverse reactions include anaphylactic shock, skin allergic reactions, injection site reactions, dizziness or headaches, nausea or vomiting, chest discomfort, numbness, and low back pain, with more than 87% of adverse reactions being mild.^27^

 A major disadvantage of ultrasound compared with MRI is that bone lesions, which are very common in rheumatologic diseases that are major causes of synovitis, such as bone marrow edema and small erosions, are only detected by MRI.^28^ Such assessments are necessary for disease staging, such as in rheumatoid arthritis, influencing treatment. However, it should be considered that MRI is not the gold standard for detecting synovitis (histological analysis is the gold standard); the use of gadolinium contrast and evaluation of multiple joints is time-consuming and expensive for routine use.^29^ In addition, the presence of metals in the body (such as prostheses or pacemakers) can interfere with MRI results, rendering the examination unfeasible for certain patients. 

 Given the similarity in accuracy and sensitivity between CEUS and contrast-enhanced MRI, and because both have 100% specificity for synovitis, combined with the greater accessibility and lower cost of ultrasound, one could envision a new direction for synovitis evaluation. However, further studies are needed to evaluate synovitis in rheumatoid arthritis to make CEUS compatible with contrast-enhanced MRI. This result aligns with a systematic review by Takase-Minegishi et al.,^30^ which evaluated the accuracy of ultrasound and MRI for synovitis diagnosis in 17 studies, only one of which used CEUS^13^ involving the metacarpophalangeal, interphalangeal, and knee joints, and concluded that ultrasound is a valid and reproducible technique. However, given the methodological variability and limited sample size, our findings reinforce the need for larger multicenter diagnostic accuracy studies using standardized protocols. Such studies should stratify the results by joint type and underlying disease to clarify the true diagnostic equivalence between modalities. 

 It should be noted that a significant discrepancy in the evaluation between CEUS and contrast-enhanced MRI was evidenced in the study by Wamser,^16^ which evaluated only the shoulder joint in rheumatoid arthritis. This discrepancy may be due to the ability of MRI to visualize the entire joint and deep part of the synovium regardless of the amount of body fat, whereas ultrasound can only visualize superficial articular recesses, which are not necessarily involved in cases of mild synovial joint synovitis. Another possible explanation is the difficulty in distinguishing synovitis from fluid, especially in old and long-lasting effusions when the fluid becomes hypoechoic. In this regard, MRI without contrast is also unable to differentiate between synovitis and synovial fluid, as both present the same signal intensity even in fluid-sensitive sequences, necessitating the use of gadolinium contrast. 

 Some studies did not specify which joints showed synovitis detected by CEUS and contrast-enhanced MRI; MRI evaluation did not specify whether the detected change was specifically synovitis, nor did it differentiate it from joint effusion and bone erosion, nor did it specify how many patients underwent MRI. CEUS features all the properties of a synovitis screening method, including low cost, availability, accessibility, high sensitivity, high specificity, and painlessness. Therefore, based on the results of this review, CEUS should be viewed as a potential screening tool rather than a replacement for MRI, especially until further evidence is available. 

 Future research should prioritize comparative trials including larger populations, diverse rheumatological conditions, and multicenter participation to ensure generalizability beyond the single-institution experience. These studies should specify the joints evaluated, the diseases of each patient, and the clinical stage of the underlying condition. Furthermore, diseases causing synovitis, such as ankylosing spondylitis, were not evaluated in these studies.^31^ Only one study^12^ evaluated osteoarthritis, the most common joint disease in people over 65 years of age, and a cause of synovitis,^32^ indicating a need for additional studies regarding this disease. Evaluation of synovitis during the treatment of the underlying disease, especially rheumatoid arthritis, should also be considered, given its significant prevalence in the population and high treatment costs. Our systematic review identified the need for new clinical trials to evaluate joints with limited research or for which research still lacks definitive results, as seen with the hand and wrist joints, which were evaluated by only two studies without specifying their data individually.^14,15^ The knee was the only joint with individualized data that showed good detection rates with both CEUS and contrast-enhanced MRI. Nonetheless, the hand and wrist joints are the most affected joints in many rheumatological diseases, such as rheumatoid arthritis.^33^

## CONCLUSION

 CEUS has significant potential as an essential tool for the early diagnosis of synovitis in clinical practice. Both contrast-enhanced microbubble ultrasonography and contrast-enhanced MRI demonstrated comparable diagnostic accuracies for synovitis, with values of 87% and 87.7%, respectively. Although these methods showed similar overall diagnostic performances, specific data for joint evaluation were only available for the knee, where CEUS outperformed MRI with a detection rate of 93.8% versus 82.9%. When assessing synovitis across different joints and underlying diseases, contrast-enhanced MRI achieved a slightly higher detection rate (88.3%) than ultrasonography (81.9%). However, these differences were not statistically significant and should be interpreted with caution, considering the small number of heterogeneous studies included. Given its portability, low cost, and safety profile, CEUS may be a feasible adjunct to MRI for routine evaluation and longitudinal follow-up of synovitis, particularly in outpatient or resource-limited settings. However, the current evidence remains limited. Larger multicenter diagnostic accuracy studies with standardized protocols are warranted to confirm the reproducibility and generalizability of these findings, particularly across different joints and rheumatological diseases. 

## Data Availability

Data supporting the findings of this study are available from the corresponding author, Márcio Luís Duarte, upon request.
